# *Saccharomyces cerevisiae* as a Model for Reprogramming of Eukaryotic Cells: Implications for the Study of the Relationship Between Metabolism and Inflammation in Chronic Disease

**DOI:** 10.1007/s12013-025-01844-w

**Published:** 2025-08-18

**Authors:** Neill S. Friedman, Glirstar J. De Britto, Alexander N. Lehner

**Affiliations:** 1 Centre for Physical Activity and Life Sciences, University of Northampton, University Drive, Northampton, NN1 5PH UK; 2 The BERG F, Northampton, UK; 3 BSc Honours Biochemistry programme, University of Northampton, Northampton, UK

**Keywords:** Mitochondria, Inflammation, Membrane potential, Fermentation, Reprogramming, Coupling efficiency

## Abstract

Inflammation is a fundamental feature of many diseases. It is part of a programmed response to threats concerning an organism’s integrity. Programming is modified by the environment and is made up of complex relationships between regulating mechanisms of metabolism. In this study, *S. cerevisiae* were used to establish a model of reprogramming, utilizing in this case a 23-h water-only fast compared to a standard high glucose environment. Crude mitochondrial preparations were made using differential centrifugation. Pyruvate Dehydrogenase Complex (PDC) activity was approximated via an assay measuring changes in ability to produce NADH. Experiments with lipopolysaccharide (LPS) involved a procedure exposing the yeast to LPS (100 ng/ml) for 90 min prior to mitochondrial isolation. Oxygen consumption rates were measured using a Clark type electrode setup. Results suggest that fasting in water can reprogram yeast mitochondria. Mechanisms modified by this process appear to regulate the ability of the mitochondria to maintain the relationship of oxygen consumption (indicative of electron transport) to RCR (indicative of membrane potential), largely separate to ATP synthesis. Although the ADP/O may be lower in the progeny of the fasted yeast, it is the fact that it maintained a higher RCR with the same or lower ADP/O, that is the important observation. Based on estimations of PDC activity, the progeny of the high glucose exposed yeast appeared less able to readily utilize pyruvate for respiration. In addition, the LPS challenge also revealed possible changes in immune response that may be resulting from glucose toxicity. In conclusion, *S. cerevisiae* can be reprogrammed to metabolically respond differently to a specific environment. This includes both a high glucose environment and a high glucose environment containing LPS (a pathogen associated molecular pattern), with regard to bioenergetic changes. These changes are associated in mammalian cells with the switch to a proinflammatory and proliferative metabolic state, analogous to that of M1 macrophages (decreased OxPhos and lower RCR), seen in atherosclerosis and other conditions. This data supports the use of this model for further investigation of proinflammatory processes and potential interventions to restore proper regulation of immune responses.

Health, being the integrity of the organism, is dependent on what, in multicellular organisms, is referred to as the immune system. But all cells, in what is known as cell autonomous immunity [1] have responses to the same three threats that define the remit of the mammalian immune system. These threats are (1) pathogens, recognized by cells as pathogen associated molecular patterns (PAMPs), (2) damage, recognized by cells as damage associated molecular patterns (DAMPs), and (3) poisons, which we argue is recognized by excessive pressure on bioenergetics supply and demand mechanics e.g. Cyanide blocks supply while dinitrophenol (DNP) exacerbates demand, and tetrodotoxin results in oxygen deprivation. In all cells, these responses require a change in metabolism, and in eukaryotic cells this is dependent on the programming of how coupling efficiency of oxidative phosphorylation within the mitochondria is regulated.

It is important to distinguish between the responses and the programming that enable those responses. In this paper it is being proposed that the various components that regulate coupling efficiency are infinitesimally adjustable and how they are ‘set’ at any given time determines how the mitochondria, cell, tissue, and so on respond to their environment (i.e., programmed). The regulators in this context are seen like valves, i.e. each regulator (e.g. AMPK, PDC, aconitase) operates with a continuous range of settings instead of like a binary switch. It would not take many of these ‘valves’ to produce virtually infinite programs, creating a basis for individual uniqueness. The acute response to stimuli such as a pathogen or poison is an adaptive response that would normally ‘reset’ back to a range commensurate with other ‘settings’ once the stimuli was dealt with, to ensure supply and demand are adequately matched. In this context, the term ‘reprogramming’ is utilized to indicate that in theory, instead of toggling single pathways, many if not all settings have been recalibrated to ensure supply and demand are matched, at the same or more likely a different set point, with a different constellation of ‘valve settings’, such that future effectiveness to respond to a particular stimuli will be altered, as will the ability to reset. Over the longer term this reprogramming would hypothetically allow for persistent adaptation to new environments.

A main focus of disease research for all the leading causes of morbidity and mortality is inflammation [2]. Inflammation refers to the mechanism by which the body recruits specialized immune cells to deal with threats to the integrity of the body while altering behavior of the organism to preserve life. It is characterized by four features: (1) redness (2) heat (3) swelling and (4) pain. Although there is overlap between the causes and consequence of these features, they are mainly due to cytokines [3] and vasodilation [4]. And the synthesis and release of pro-inflammatory cytokines is dependent on changes in cellular metabolism [5].

The M1 and M2 macrophage phenotypes are characterized by their bioenergetic states. M1 macrophages demonstrate a greater dependence on glycolysis to match their energy supply and demand while M2 macrophages show a greater reliance on oxidative phosphorylation to match their energy requirements [6]. M1 and M2 states result in the upregulation of pro and anti-inflammatory cytokines, respectively [6]. We propose that sensitivity to changes in nutrient status acts as a trigger for cells to switch from the proinflammatory state to the anti-inflammatory state, and that chronic glucose exposure programs cells to be desensitized to the drop in energy supply, driving chronic inflammation and potentially what some have called the proinflammatory cytokine storm (for reviews and history of the term see [7, 8]).

In line with the above, pro-inflammatory signaling is a healthy response to threats against the body. The body, however, can be reprogrammed by its environment to be more or less sensitive to the signals of threats, and this often involves lipids. For when the integrity of cells or tissues are threatened, the cells have evolved mechanisms to repair and rebuild. At the most fundamental level, repairing or rebuilding cells and tissues requires lipogenesis for components of the plasma membrane and more. The organism therefore needs to prepare to heal after battling and this therefore ties immune responses to lipogenesis, whereby the reverse, forcing lipogenesis, produces proinflammatory signaling. This is seen in itaconate which, in response to PAMPs, cuts off the Krebs Cycle and electron transport chain and initially promotes fermentation [9] and citrate accumulation which contributes to increased flux through the pentose phosphate pathway and to de novo fatty acid synthesis, respectively. In many instances lipids also act as sensors of oxidative stress [10]. Oxidation of cardiolipin for example precedes the lowering of the membrane potential [11] and arguably facilitates it while the increase in superoxide downregulates supply available for oxidative phosphorylation e.g., through the inactivation of aconitase [12] which redirects resources to de novo fatty acid and cholesterol synthesis. At the same time, this forces the cell toward fermentation, regardless of oxygen availability (Warburg/Crabtree effect).

What remains unclear is how to determine if the cells of the body are in fact programmed by their environment to be over- or under-sensitive to oxidative signals, and once determined, how to reprogram them to produce nonpathological tissues and organs. We have previously looked at reprogramming in rats and kidney function [13], in research related to fetal programming and hypertension. This, and our work around it, highlighted that this reprogramming is complex, often works in a biphasic manner, and at the level of mammals (which would also include humans) takes time on the order of months or years.

The yeast *S. cerevisiae* is crab-tree positive, meaning that it has been shown to switch from fermentation to respiration when glucose in the surrounding media is depleted [14]. In other words, it is a facultative aerobe that ferments when in high glucose environments, like humans do (particularly evident in cancer cells [15]). Despite some differences, such as not having a proton pumping Complex I [16], the crab-tree positive nature of *S. cerevisiae* along with its highly conserved mitochondrial respiratory chain has been identified as an excellent model organism for investigating mitochondrial behavior [17]. The research presented here therefore set out to determine if *S. cerevisiae* could be used as a model of persistent mitochondrial reprogramming across multiple generations (cellular divisions). And once established, it was tested whether it could be used as a model of reprogramming of immune responses analogous to those that lead to chronic inflammation, such as that seen in cardiovascular disease.

## Materials and Methods

### Yeast Culture

BY4742 yeast (Invitrogen), from the original stock were streaked on a YPG agar plate, (Agar [OXID], 1%w/v, yeast extract [OXID], 2%w/v bacterial peptone [OXID], 3% v/v glycerol) and incubated at 30 °C for 24-h. A single robust colony was selected and added to 2 ml YPD broth, referred to here as high glucose (HG) broth (1%w/v yeast extract [OXID], 2%w/v bacterial peptone [OXID], 2% w/v α D-Glucose [Aldrich]) and incubated (INCU-Shake FL18-750R) for 24-h at 30 °C, rotated at 200 rpm. After which 20 μl was aliquoted on to a YPG agar plate which was incubated at 30 °C for 24 h before being placed at 4 °C. All experimental cultures were started using a single colony of SC selected from this plate.

### Reprogramming Experiment

A single yeast colony was transferred into 2 ml HG broth in a 15 ml Falcon tube and warmed for three hours in a shaker at 30 °C/200 rpm. This was then transferred to a 500 ml glass flask containing 100 ml HG broth and returned to the incubator for 24 h at 30 °C 200 rpm.

After 24 h the yeast were centrifuged (Eppendorf, 5804 R**)** at 4 °C/1000xg/5 min then resuspended in 30 ml sterile cold _d_H_2_0 and re-pelleted by centrifugation at 4 °C/1000xg/5 min before being resuspended in 5 ml sterile _d_H_2_0. A cell count reflecting greater than 8 × 10^9^ live cells at this stage was seen to produce sufficient mitochondria preparations. Equal amounts of yeast were added to either fasted in 100 ml sterile _d_H_2_O (F) or to 100 ml HG (HG) and incubated at 30 °C 200 rpm for 23 h.

Both conditions were pelleted by centrifugation at 4 °C/1000 g/5 min before being resuspended in 7 ml HG broth and placed on ice. **It is important to recognize that a matching number of progenitor cells from each condition was seeded into 500** **ml HG broth** (1 × 10^9^ was found to be the minimum number of cells needed for each intervention to result in a sufficient mitochondrial yield after isolation). Each 500 ml was divided equally (100 ml each) into 5 × 500 ml flasks and incubated at 30 °C/200 rpm for 20 h. Yeast progeny were pelleted, washed with _d_H_2_O, and combined to produce a single pellet for each condition.

### LPS Challenge

Yeast challenged with LPS were conducted on separate cultures to those that remained unchallenged (due to timing constraints). Unchallenged cultures progressed directly to isolation. Yeast from both conditions (F and HG) were washed in 4 °C _d_H_2_O, resuspended in 50 ml HG broth with LPS (100 ng/ml), and placed into 250 ml flasks with LPS, concentration based on the work of Marques et al. [18]. After 90 min the yeast were pelleted, washed and re-pelleted the same as the unchallenged yeast.

### Crude Mitochondrial Preparation

Yeast isolation followed the protocol for isolating yeast mitochondria, as described by Gregg et al. [19], with a few key notable exceptions. As all pellets were combined, we picked up the original protocol at step 5. All pelleting of yeast prior to step 17 was done at 4 °C/1000 *g*/5 min. At step 14 rather than powdered Zymolyase, the enzyme was dissolved in _d_H_2_O, aliquoted, and stored at −70 °C. At the final step, the isolated mitochondria were suspended in 0.1 M MES/NaOH buffer, pH 6.5. The protein concentration of the final mitochondrial suspension was assayed using the Biuret protein assay, with BSA as protein standard.

### Respirometry Assay

Analysis, of respiration rate and proton leak kinetics, of the isolated mitochondria, was conducted using a Rank Brothers Clark Electrode Setup (oxygen sensitive electrode), connected to an AD Instruments PowerLab and LabChart 8 software (AD Instruments).

The Clark Electrode chamber (3.5 ml) with recirculating water jacket was regulated at 30 °C and was calibrated using electrode buffer (20 mM Tris/HCl, 450 mM sorbitol, 100 mM KCl, 0.5 mM EGTA, 5 mM MgCl2, 10 mM K2HPO4, 0.1% defatted BSA, pH 6.8), assumed to contain 424.8nmol 0_2_/ml at 30 °C [20, 21]. Once calibrated the chamber containing 3.5 ml electrode buffer was carefully closed, making sure to be free of air bubbles, before 60 μl of 100 mM d-Lactate, was added using a micro-syringe through a small aperture. d-Lactate is an electron donor to cytochrome c in yeast mitochondria [21, 22].

Once the electrode trace had stabilized, the mitochondrial prep (F, HG, F-LPS or HG-LPS), was added to a final in-chamber concentration of 0.3 mg/ml. Then 5 μl of 25 mM Adenosine Diphosphate (ADP), to establish State 3. Then 2 μl of 0.01 mM Oligomycin A (OMY), an ATP synthase inhibitor, was added to establish State 4_O_. 2 × 2 μl titrations of 1 mM Carbonyl cyanide-p-trifluoromethoxy phenylhydrazone (FCCP), an ionophore that acts as an uncoupling agent, used to establish the maximal, uncoupled oxygen consumption rate (OCR). 1.5 μl of 3.5 mM Antimycin A (Anti A), a Complex III inhibitor, was used to establish residual/non-mitochondrial oxygen consumption (ROX).

The electrode chamber was thoroughly cleaned before and after each experimental run. At the end of each day, a few crystals of Sodium Dithionite were sprinkled in the open chamber, removing all oxygen. This was used to re-verify the calibration, by checking if the LabChart trace dropped to zero.

### Data Analysis of Respirometry

Each condition was assayed in duplicate in three independent experiments. The real time electrode data was captured using LabChart 8 analysis software, and the traces later analyzed using the Data Pad functionality. This data was then exported to an Excel spreadsheet, where the data was normalized for the protein concentration and the voltage fluxes converted to OCR measured in nmol of O_2_ consumed per minute per mg protein (nmol/min/mg) this was done using the following formula:

$${\rm{OCR}}({\rm{nMol}}/\min /{\rm{mg}})=\frac{\left({{\rm{O}}}_{2}{\rm{slope}}\left\{{\rm{units}}\right\}\times 60\right)\times 424.8}{{\rm{Oxygen}}\; {\rm{full}}\; {\rm{chart}}\; {\rm{span}}\left\{{\rm{units}}\right\}\times {\rm{Concentration}}\; {\rm{of}}\; {\rm{protein}}\; {\rm{in}}\; {\rm{chamber}}\left\{{\rm{mg}}/{\rm{ml}}\right\}}$$

OCR was then calculated for State 3, State 4, State 4_O_, and Maximum OCR by deducting the ROX value from the values derived. This can be done because the ROX value is assumed to be constant within this setup [23]. These can be seen as presented in Supplementary Fig. 1.

ADP/O ratio was determined by identifying the duration (s) of ADP consumption from when the slope changed following the addition of ADP until the slope returned to the pre-ADP value. By subtracting the amount of oxygen consumed by leak (calculated from State 4_O_ OCR) from the amount consumed for ATP production (calculated from State 3 OCR), by dividing each OCR by 60 and multiplying by the duration, we were able to determine the nmol O2 consumed and divide that into the nmols of ADP consumed.

### Pyruvate Dehydrogenase Complex (PDC) Activity Based on NADH Production

The assay was done on a 96-well plate with 18 reaction wells and 18 negative controls with no NAD^+^. Each reaction well contained 40 micrograms protein of mitochondrial sample brought up to 25 μl with homogenization buffer with no BSA. 25 μl of 0.25% v/v Triton X-100 was added to lyse the mitochondrial membranes and incubated at room temperature for 10 min after a quick mix with plate mixer. 100 μl of assay buffer (50 mM K3PO4, 1 mM MgCl2, 2 mM pyruvate, 2.6mM L-Cysteine, 2 mM Thiamine pyrophosphate, and 2.5 mM NAD+ (SIRT substrate), pH 7.2) was added and incubated at 30 °C for 10 min after a quick mix with plate mixer. 100 μl of coenzyme A (final concentration 0.13 mM) was added to start the reaction and a quick mix was given by reverse pipetting with a multi-channel pipette. Absorbance readings were taken at 340 nm every 60 s for 10 min with an endpoint set up using microplate reader (Bio-Rad 680 XR).

### Statistical Analysis

All statistical analyses were conducted using Microsoft Excel. Measurements were done in duplicate (with the exception of PDC). Error bars show the Standard Error of the Mean (S.E.M.) of averages from three independent experiments.

When examining statistical significance, 1-tailed Paired Student T tests were used when comparing data between F and F-LPS as well as between HG and HG-LPS and 1-tailed unpaired Student T tests were used when comparing F or F-LPS to either HG or HG-LPS, in these cases equal variance was assumed.

To determine statistical significance of the percentage changes as a result of the LPS challenge in each of the F and HG conditions, the following steps were taken: first the values from each repeat of F was divided by the mean value of the same data from F-LPS, providing the 1^st^ value set. Then the value from each of the F-LPS repeats was divided by the mean value of the same data from the F, providing the 2^nd^ data set. A 1-tailed paired Student T test was then conducted between the two data sets. The same process was followed to determine the significance of this data between HG and HG-LPS.

As this is exploratory research to justify further research, *p* ≤ 0.10 was considered statistically significant.

## Results and Discussion

Our aim was to determine if fasting of *S. cerevisiae* would change their mitochondrial response to a high glucose environment (which they are typically grown in) and to an LPS challenge. In these initial experiments being present here, we investigated whether key respiratory parameters were set to respond differently to the same growth environment. Specifically, after growing in a high glucose (2%) broth, we looked at State 3 and State 4_O_ respiration as well as their ratio known as the Respiratory Control Ratio (RCR) as an indicator of coupling efficiency [24], and the difference between Uncoupled Maximum OCR and State 3 (often referred to as Reserve Capacity). Importantly, we also looked at the ADP/O ratio, to determine how much oxygen was consumed to respond to a given amount of additional ADP, as a different indicator of coupling efficiency [24]. We hypothesized that the progeny of the fasted yeast would have been reprogrammed to be more tightly coupled (higher efficiency) and more metabolically flexible, ‘tilting’ more toward oxidative phosphorylation than toward fermentation, even in the same environment. We therefore also set out to determine if reprogramming altered PDC activity, facilitating additional oxidative phosphorylation. Theoretically, this would be akin to restoring health after a threatening experience.

**Fig. 1:**
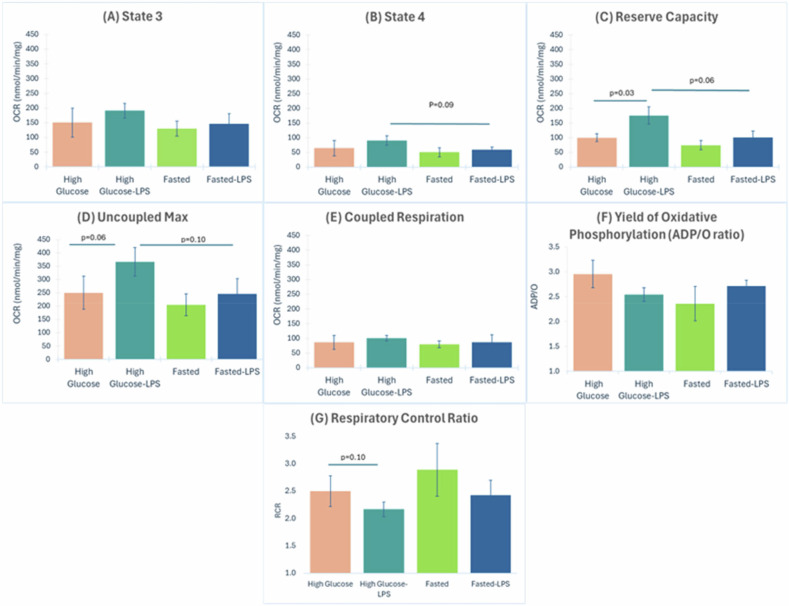
Variations in oxygen consumption rate (OCR) characteristics of the mitochondria isolated from the progeny of the pre-conditioned yeast *S. cerevisiae*. **A** State 3 respiration determined from the maximal oxygen consumption slope immediately post ADP addition. **B** State 4_O_ respiration determined measuring the slope after the addition of Oligomycin A (known to block ATP synthase). **C** Reserve Capacity, determined as the difference between the Uncoupled Max and State 3. **D** Uncoupled Maximum respiration, determined using the uncoupler FCCP at a concentration pre-determined to achieve maximum uncoupling without inhibiting respiration. **E** Coupled respiration, determined by deducting State 4_O_ respiration from State 3 respiration. **F** Yield of Oxidative Phosphorylation (ADP/O ratio). Calculated by dividing the added 125 nmol ADP by the nmols of oxygen consumed, excluding oxygen accounted for by State 4_O_, for the duration of added ADP consumption. **G** RCR, determined by dividing State 3 by State 4_O_. Good coupling of oxidative phosphorylation is reflected by a high RCR [24]. (1-tailed Student T tests were performed to test for significance, paired when comparing data from the same conditioning and unpaired, assuming equal variance, when comparing across the two conditions). Each sample was assayed in duplicate and the results are the mean of three independent experiments ±S.E.M

It was clearly evidenced that there were changes in the way the mitochondria utilized fuel in relation to the rate at which they consumed oxygen. Comparing the progeny of the fasted yeast with the progeny of the non-fasted HG yeast (both progenies having grown in normal high glucose broth), there was a trend toward a lower OCR in the progeny of the fasted yeast than in the ‘normal’ high glucose progeny. When exposed to LPS, the differences between the two groups were most convincing for State 4_O_, reserve capacity, and uncoupled max OCR (Fig. 1).

**Fig. 2:**
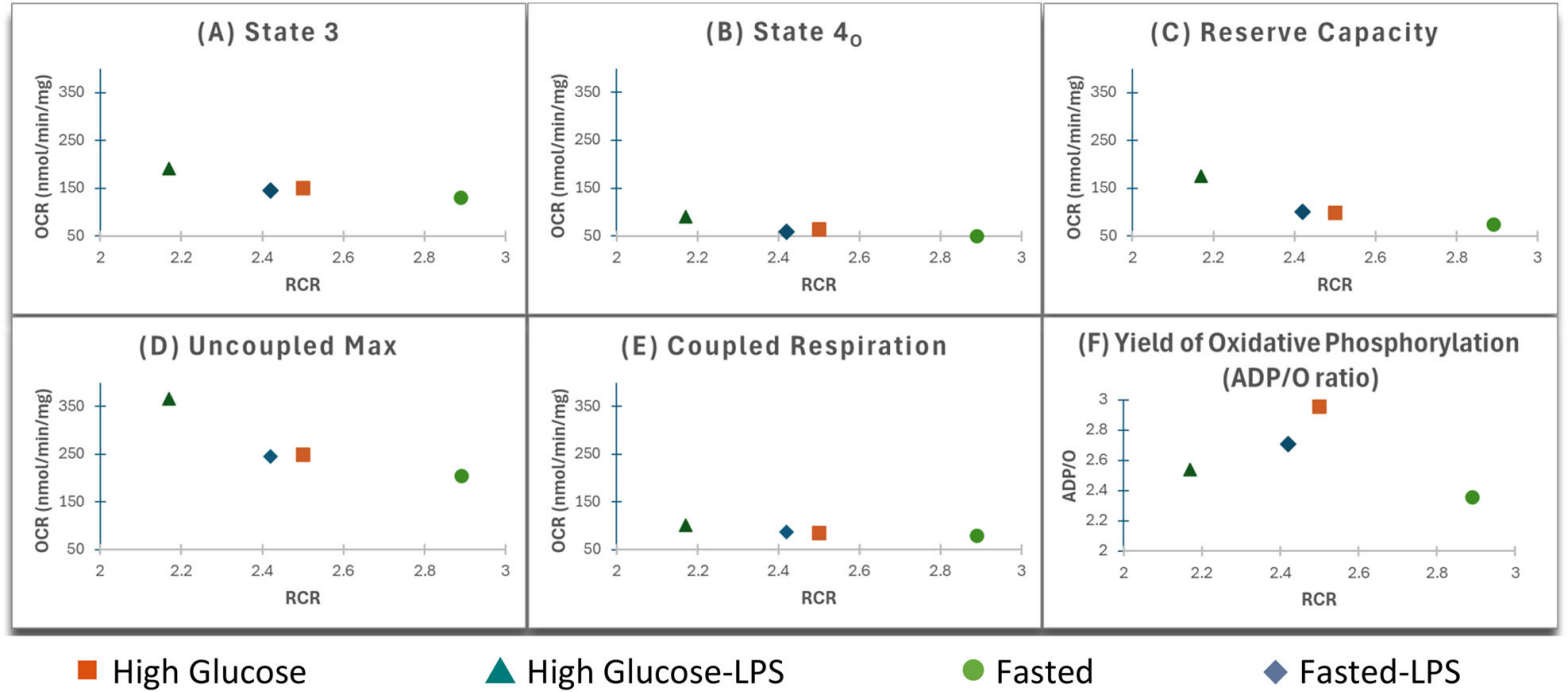
Data shown in Fig. 1 relative to the RCR (State 3/State 4_O_). **A** State 3 respiration, determined from the maximal oxygen consumption rate immediately post ADP addition, **B** State 4_O_ respiration determined measuring the slope after the addition of Oligomycin A (known to block ATP synthase), **C** Reserve Capacity determined as the difference between the Uncoupled Max and State 3, **D** Uncoupled Maximum respiration, determined using the uncoupler FCCP at a concentration pre-determined to achieve maximum uncoupling without inhibiting respiration, **E** Coupled Respiration, determined by deducting State 4_O_ respiration from State 3 respiration, **F** Yield of Oxidative Phosphorylation (ADP/O ratio). Calculated by dividing the added 125 nmol ADP by the nmols of oxygen consumed, excluding oxygen accounted for by State 4_O_, for the duration of added ADP consumption.

LPS was not as clearly seen to affect State 4_O_ of the F progeny as it was seen to increase State 4_O_ of the HG progeny. If considering the RCR however as an indicator of coupling efficiency (and by extension an inexact indicator of membrane potential), when the F progeny were exposed to LPS, their State 4_O_ respiration was possibly achieving roughly the same OCR at a lower membrane potential (Fig. 2B). That could mean that in response to LPS, the membrane integrity was maintained, and proton leak was decreased (for example possibly due to closing or possible turnover of a potential uncoupling protein (e.g., _SC_MUC [25, 26]) turnover or the block in supply reduced ROS production diminishing activation of adenine nucleotide translocators [10, 27]). While the difference in State 3 of the F progeny after LPS exposure was not as great as the difference observed in the HG progeny after LPS exposure, it was also not to the same degree proportionally because the mitochondria needed more oxygen to make the same amount of ATP (Fig. 2F) This could for example possibly be due to a change in the electron transport chain (ETC). State 4_O_ is when ATP synthase is inhibited and is often thought to be an indicator of membrane integrity. However, the possibility that different programs can achieve the same OCR at different membrane potentials could reveal a new understanding of how the regulation of coupling efficiency is programmed.

LPS caused Reserve Capacity (i.e., Uncoupled Max – State 3) to be higher in both progenies, but more so in the HG Progeny (Fig. 1C). Being higher in the HG progeny possibly suggests that the F progeny were programmed to be more tightly coupled, and the higher RCR in relation to the higher OCR due to LPS exposure for the F progeny is more clearly seen in Fig. 2C. Based on how high the LPS caused the Uncoupled Max to be in the HG progeny (Fig. 2D), it appears the inefficiency that is possibly built into the ETC of the HG progeny is the main contributor to the observed high Reserve Capacity (which might be thought of as greater ‘slippage’ which could have implications for what Reserve Capacity really tells us).

A key to making sense of this is that Coupled Respiration (Fig. 2E) had virtually the same OCR for all conditions, i.e., reprogramming did not affect OCR via a change in the difference between State 3 and State 4_O_ (of a particular cell population) when dealing with a high glucose environment. Nor did it clearly affect this difference when challenged with a PAMP (although there was a trend toward LPS exposed cells to have a greater difference). Yet, what is clearly different is that programming possibly caused them to make adjustments such that they maintained the same difference between State 3 and State 4_O_ OCR with different RCR’s (State 3/State 4_O_, which is thought to indicate different membrane potentials). We believe this is fundamental to understanding what is happening. And will explore this further with real time quantitative measures of membrane potentials.

The RCR on its own suggested tighter coupling in the F progeny, and this is in agreement with studies on fasted trout [24]. And in both F and HG progenies LPS caused a decrease in the RCR, but it was a greater decrease in the F progeny (19% compared to 15%; Supplementary Table 1), This is also quite striking when considered with the ADP/O ratio. Both RCR and ADP/O say something complex about coupling efficiency, and therefore, looking at how they differ can be quite revealing. LPS caused ADP/O to be slightly higher in F progeny, yet slightly lower in HG progeny. This once again supports the fact that the protocol has in fact reprogrammed the yeast. This also supports the conclusion that this reprogramming resulted in the F progeny being more tightly coupled. However, the response with regard to ADP/O unlike the RCR suggests the F progeny became more efficient, using less oxygen to utilize the ADP in response to LPS. And this becomes very clear when ADP/O is plotted against RCR (Fig. 2F), where the LPS appears to make the F progeny more like the non-LPS HG Progeny. But then the increase in oxygen consumption in the HG progeny after LPS is not matched to the ADP utilization comparably to the F progeny (i.e. ATP production to meet demand may be hampered). Based on our previous work in mammalian tissues [13] where we first proposed that programing of an organism is the adjusting of the regulation of coupling efficiency, we might speculate that the HG progeny are programmed to not be able to maintain their ability to utilize ADP from an increased oxygen consumption at lower membrane potential. We have called this ‘coping capacity’, and it appears that the reprogrammed F progeny are possibly able to better deal with the toxic high glucose environment and still be able to adjust to the toxic high glucose + LPS environment possibly by becoming less efficient with regard to ETC and membrane integrity dynamics but more efficient with regard to ATP synthesis; perhaps simultaneously lowering membrane potential and increasing OCR (which is classic uncoupling), while the HG progeny were already operating at a lower efficiency, their membrane potential was possibly even lower after LPS exposure. From a practical sense, whereas in our animal models we were able to predict relative proximity to death, this data does not reveal which progeny would be better off if exposed to the *E. coli* that the LPS came from. But from what we have seen in this study, it will now be exciting to move to the next phase and use the model to actually relate these changes to membrane potential.

**Fig. 3:**
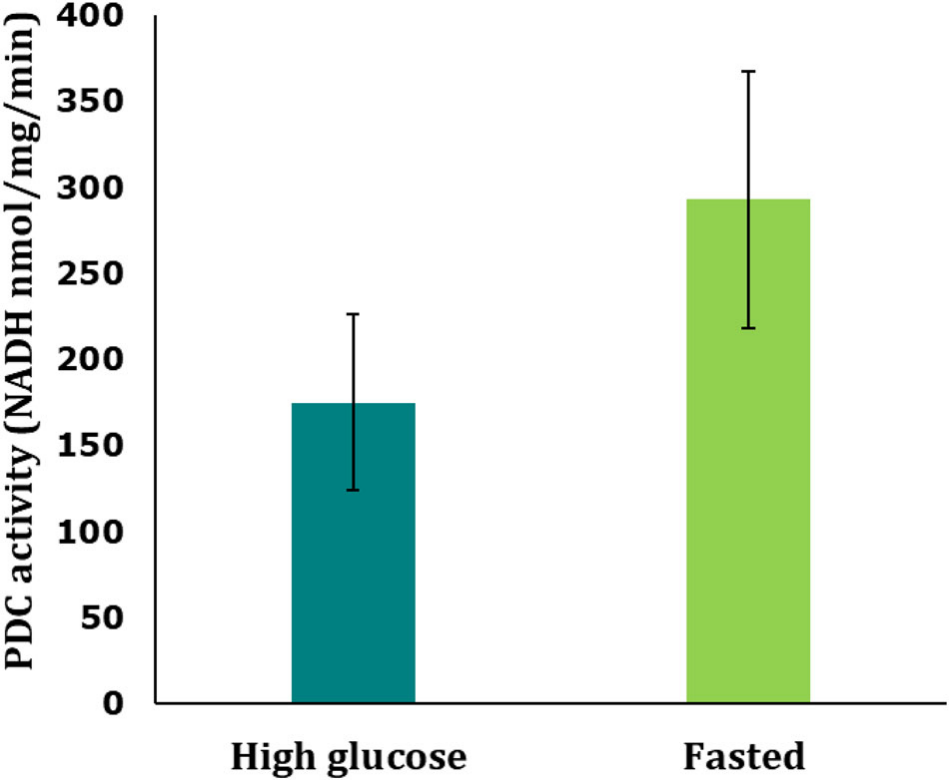
Pyruvate Dehydrogenase Complex (PDC) activity based on NADH production. High Glucose (blue) and Fasted (green) mitochondria isolated from cells regrown in high glucose after nutritional interventions. The PDC activity levels are represented as moles of NADH produced per milligram of protein per minute (nmol/mg/min). 1 unit of PDC is considered to form 1µmol of NADH per minute at pH 7 and 30 °C for high glucose and fasted mitochondria. (1nmol/mg/min = 1 unit/mg). Data represent the single measurements from three independent experiments ±S.E.M. Mitochondria were frozen at −70 °C and the assay was carried out on a single day. Negative controls with no NAD^+^ in the assay buffer were used which showed no increase in absorbance readings over the reaction time. The reaction wells were blanked with negative control wells to find the actual rise in absorbance due to NADH levels

The PDC data (Fig. 3) supports the idea that the reprogramming could be affecting fuel supply, to direct the glucose metabolite, pyruvate, towards the Krebs cycle and oxidative phosphorylation (more ‘opened valve’), whereas cells that had evolved from progenitors that had always been in high glucose were more restrictive on allowing glucose metabolites access to the mitochondria, tilting toward fermentation (more ‘closed valve’). In humans it is thought that being too heavily geared toward fermentation could contribute to cytokine storms (e.g. in conditions like sepsis or Covid19 [28]) or just chronic inflammation (e.g. in conditions like atherosclerosis or asthma), as the cells cannot shift back to oxidative phosphorylation in response to subtle signals by nutrient/energy sensors. Tilting towards fermentation would theoretically also lead epithelial cells to proliferate, like in cancer or endometriosis.

The data from the PDC experiments (Fig. 3) showed a great deal of variation due to one of the preparations producing anomalously lower absorbance readings. We were unable to determine what was different, so the data was included in the results and although it increased the variance, it still did not eliminate the average difference between the two groups which is why we are reporting it as we are (*p* = 0.32). However, all three independent experiments resulted in the PDC activity being higher in the progeny of the fasted yeast. And when the anomalous data was removed from analysis, there was no overlap between the data sets, further suggesting the difference was real. Further research needs to be done to confirm this. However, the assay contains triton-x and due to technique, this sometimes led to the formation of bubbles in the wells, which was eventually resolved by using a small gauge needle. It is possible that this had not yet been perfected, and some bubbles were missed.

Yeast, as a model of immune response, is a challenging idea. It has been proposed that yeast started producing ethanol when plants started producing fruit that fell to the ground with toxic levels of sugar [29]. The history of ethanol fermentation positions the process as having the core parts of our immune system built into our single cell ancestors that we share with fungi, with ethanol secretion in the yeast, and fermentation in general, as a defensive (immune/pathogen) response conjoined to glycolysis as a detoxifying (immune/poison) response, and the pentose phosphate pathway as a fortifying (immune/damage/pathogen/poison) response.

Variation between progeny of the F vs HG in this experiment is thought to show reprogramming of how coupling efficeincy is managed. And the variation in LPS treated samples is thought to indicate reprograming of a cellular immune response. Programming, as adjustments of the regulation of coupling efficiency [13], determines how much fuel and/or how much oxygen is required to meet a specific demand and at what membrane potential the mitochondria make that transaction. This supply and demand adjustment programming then plays out in all cellular processes. Based on work in Martin Brand’s group over more than three decades and more recently in very small mammals [30, 31], the ability to manage the matching of supply and demand at a lower membrane potential could facilitate a longer life.

### Conclusion

In summary, we first demonstrated that *S. cerevisiae* could be reprogrammed to metabolically respond differently to a specific environment, compared to ‘normally’ programmed yeast. To our knowledge, this is the first time it has been shown that programming, at the level of coupling efficiency, could be altered over generations by dietary changes, with reprogramming the regulation of coupling efficiency being the novel contribution. Furthermore, the reprogrammed yeast were also shown to respond differently to a PAMP (LPS), with regard to bioenergetic changes associated in mammalian cells with the switch to a proinflammatory and proliferative metabolic state. This switch from ‘tilting’ toward oxidative phosphorylation to ‘tilting’ toward fermentation, is analogous to that of M1 macrophages or endothelial cells seen in atherosclerosis [32], as well as possibly all mitochondria containing mammalian cells when exposed to excess glucose. Research that better delineates glucose poisoning at the cellular and organismal level could also have a foundation here. This data clearly supports the use of this model for further investigation of inflammatory processes and potential interventions to restore proper regulation of immune responses, and thereby restore coherence, the true basis of health. A key issue highlighted by this initial study is that accurate quantitative measures of membrane potential will be of immense value in future studies. At this stage, what this study contributes to the longstanding, even ancient, concept of energy imbalance as a cause of pathology and death, is a clear experimental model and the language needed to formalize the idea and address the problem of how to reprogram health.

## Supplementary information

Supplementary Material

## Data Availability

All data underpinning this publication are openly available from the University of Northampton Research Explorer at 10.24339/574e7e3c-3193-443b-9fa0-b8eed6b621b1.

## References

[1] Howard, J. C. (2007). Introduction: cell-autonomous immunity. *Microbes and Infection*, *9*(14), 1633–1635. 18024123 10.1016/j.micinf.2007.09.003

[2] Furman, D., Campisi, J., Verdin, E., Carrera-Bastos, P., Targ, S., Franceschi, C., Ferrucci, L., Gilroy, D. W., Fasano, A., Miller, G. W., Miller, A. H., Mantovani, A., Weyand, C. M., Barzilai, N., Goronzy, J. J., Rando, T. A., Effros, R. B., Lucia, A., Kleinstreuer, N., & Slavich, G. M. (2019). Chronic inflammation in the etiology of disease across the life span. *Nature Medicine*, *25*(12), 1822–1832. 31806905 10.1038/s41591-019-0675-0 PMC7147972

[3] Chen, L., Deng, H., Cui, H., Fang, J., Zuo, Z., Deng, J., Li, Y., Wang, X., & Zhao, L. (2017). Inflammatory responses and inflammation-associated diseases in organs. *Oncotarget*, *9*(6), 7204–7218. 29467962 10.18632/oncotarget.23208 PMC5805548

[4] Tiwari P. S. (2024) Basic mechanism involved in the process of inflammation and repair-I. In: S. P. Gupta, M. Gupta, (Eds). *Edited book of pathophysiology*. (p. 32–44). First. Iterative International Publishers, Selfypage Developers Pvt Ltd.

[5] Sun, L., Yang, X., Yuan, Z., & Wang, H. (2020). Metabolic reprogramming in immune response and tissue inflammation. *Arteriosclerosis, Thrombosis, and Vascular Biology*, *40*(9), 1990–2001. 32698683 10.1161/ATVBAHA.120.314037 PMC7484156

[6] Ge, T., Yang, J., Zhou, S., Wang, Y., Li, Y., & Tong, X. (2020). The role of the pentose phosphate pathway in diabetes and cancer. *Frontiers in Endocrinology*, *11*, 365. 32582032 10.3389/fendo.2020.00365 PMC7296058

[7] Fajgenbaum, D. C., & June, C. H. (2020). Cytokine storm. *The New England Journal of Medicine*, *383*(23), 2255–2273. 33264547 10.1056/NEJMra2026131 PMC7727315

[8] Nie, J., Zhou, L., Tian, W., Liu, X., Yang, L., Yang, X., Zhang, Y., Wei, S., Wang, D. W., & Wei, J. (2025). Deep insight into cytokine storm: From pathogenesis to treatment. *Signal Transduction and Targeted Therapy*, *10*, 112. 40234407 10.1038/s41392-025-02178-y PMC12000524

[9] Heinz, A., Nonnenmacher, Y., Henne, A., Khalil, M. A., Bejkollari, K., Dostert, C., Hosseini, S., Goldmann, O., He, W., Palorini, R., Verschueren, C., Korte, M., Chiaradonna, F., Medina, E., Brenner, D., & Hiller, K. (2022). Itaconate controls its own synthesis via feedback-inhibition of reverse TCA cycle activity at IDH2. *Biochimica et Biophysica Acta (BBA) - Molecular Basis of Disease*, *1868*(12), 166530. 36038039 10.1016/j.bbadis.2022.166530

[10] Azzu, V., Parker, N., & Brand, M. D. (2008). High membrane potential promotes alkenal-induced mitochondrial uncoupling and influences adenine nucleotide translocase conformation. *The Biochemical Journal*, *413*(2), 323–332. 18426390 10.1042/BJ20080321 PMC2474560

[11] Kagan, V. E., Tyurin, V. A., Jiang, J., Tyurina, Y. Y., Ritov, V. B., Amoscato, A. A., Osipov, A. N., Belikova, N. A., Kapralov, A. A., Kini, V., Vlasova, I. I., Zhao, Q., Zou, M., Di, P., Svistunenko, D. A., Kurnikov, I. V., & Borisenko, G. G. (2005). Cytochrome c acts as a cardiolipin oxygenase required for release of proapoptotic factors. *Nature Chemical Biology*, *1*(4), 223–232. 16408039 10.1038/nchembio727

[12] Gardner, P. R., & Fridovich, I. (1992). Inactivation-reactivation of aconitase in *Escherichia coli*. A sensitive measure of superoxide radical. *The Journal of Biological Chemistry*, *267*(13), 8757–8763. 1315737

[13] Engeham, S., Mdaki, K., Jewell, K., Austin, R., Lehner, A. N., & Langley-Evans, S. C. (2012). Mitochondrial respiration is decreased in rat kidney following fetal exposure to a maternal low-protein diet. *Journal of Nutrition and Metabolism*, *2012*, 989037. 22536494 10.1155/2012/989037 PMC3321454

[14] Di Bartolomeo, F., Malina, C., Campbell, K., Mormino, M., Fuchs, J., Vorontsov, E., Gustafsson, C. M., & Nielsen, J. (2020). Absolute yeast mitochondrial proteome quantification reveals trade-off between biosynthesis and energy generation during diauxic shift. *Proceedings of the National Academy of Sciences of the United States of America*, *117*(13), 7524–7535. 32184324 10.1073/pnas.1918216117 PMC7132131

[15] Warburg, O. (1925). The metabolism of carcinoma cells1. *The Journal of Cancer Research*, *9*(1), 148–163.

[16] Matus-Ortega, M. G., Cárdenas-Monroy, C. A., Flores-Herrera, O., Mendoza-Hernández, G., Miranda, M., González-Pedrajo, B., Vázquez-Meza, H., & Pardo, J. P. (2015). New complexes containing the internal alternative NADH dehydrogenase (Ndi1) in mitochondria of *Saccharomyces cerevisiae*. *Yeast (Chichester, England)*, *32*(10), 629–641. 26173916 10.1002/yea.3086

[17] Bonnefoy, N., & Fox, T. D. (2007). Directed alteration of *Saccharomyces cerevisiae* mitochondrial DNA by biolistic transformation and homologous recombination. *Methods in Molecular Biology*, *372*, 153–166. 10.1007/978-1-59745-365-3_11. 18314724 10.1007/978-1-59745-365-3_11 PMC2771616

[18] Marques, J. M., Rodrigues, R. J., de Magalhães-Sant’Ana, A. C., & Gonçalves, T. (2006). *Saccharomyces cerevisiae* Hog1 protein phosphorylation upon exposure to bacterial endotoxin*. *The Journal of Biological Chemistry*, *281*(34), 24687–24694. 16790423 10.1074/jbc.M603753200

[19] Gregg, C., Kyryakov, P., & Titorenko, V. I. (2009). Purification of mitochondria from yeast cells. *Journal of Visualized Experiments : JoVE*, *30*(30), 1417. 10.3791/1417 PMC3149909 19704406

[20] Reynafarje, B., Costa, L. E., & Lehninger, A. L. (1985). O_2_ solubility in aqueous media determined by a kinetic method. *Analytical Biochemistry*, *145*(2), 406–418. 4014672 10.1016/0003-2697(85)90381-1

[21] Esteves, T. C., Parker, N., & Brand, M. D. (2006). Synergy of fatty acid and reactive alkenal activation of proton conductance through uncoupling protein 1 in mitochondria. *The Biochemical Journal*, *395*(3), 619–628. 16451125 10.1042/BJ20052004 PMC1462701

[22] Pallotta, M. L., Valenti, D., Iacovino, M., & Passarella, S. (2004). Two separate pathways for d-lactate oxidation by *Saccharomyces cerevisiae* mitochondria which differ in energy production and carrier involvement. *Biochimica et Biophysica Acta (BBA) - Bioenergetics*, *1608*(2), 104–113. 14871487 10.1016/j.bbabio.2003.10.008

[23] Brand, M. D., & Nicholls, D. G. (2011). Assessing mitochondrial dysfunction in cells. *The Biochemical Journal*, *435*(2), 297–312. 21726199 10.1042/BJ20110162 PMC3076726

[24] Affourtit, C., Wong, H. S., & Brand, M. (2018). Measurement of proton leak in isolated mitochondria. *Methods in Molecular Biology (Clifton, NJ)*, *1782*, 157–170. 10.1007/978-1-4939-7831-1_9 29850999

[25] Morales-García, L., Uribe-Carvajal, S., Chiquete-Felix, N., & Espinoza-Simon, E. (2019). The reversible opening of ScMuc demonstrates a high potential as a cellular protection system. *Biophysical Journal*, *116*(3), 419a.30658838

[26] Morales-García, L., Ricardez-García, C., Castañeda-Tamez, P., Chiquete-Félix, N., & Uribe-Carvajal, S. (2021). Coupling/Uncoupling reversibility in isolated mitochondria from *Saccharomyces cerevisiae*. *Life (Basel)*, *11*(12), 1307. 34947838 10.3390/life11121307 PMC8707985

[27] Brand, M. D., Pakay, J. L., Ocloo, A., Kokoszka, J., Wallace, D. C., Brookes, P. S., & Cornwall, E. J. (2005). The basal proton conductance of mitochondria depends on adenine nucleotide translocase content. *The Biochemical Journal*, *392*(Pt 2), 353–362. 16076285 10.1042/BJ20050890 PMC1316271

[28] Meng, Q. F., Tian, R., Long, H., Wu, X., Lai, J., Zharkova, O., Wang, J. W., Chen, X., & Rao, L. (2021). Capturing cytokines with advanced materials: a potential strategy to tackle COVID‐19 cytokine storm. *Advanced Materials (Deerfield Beach, Fla.)*, *33*(20), 2100012. 33837596 10.1002/adma.202100012 PMC8250356

[29] Dashko, S., Zhou, N., Compagno, C., & Piškur, J. (2014). Why, when, and how did yeast evolve alcoholic fermentation? *FEMS Yeast Research*, *14*(6), 826–832. 24824836 10.1111/1567-1364.12161 PMC4262006

[30] Porter, R. K., & Brand, M. D. (1993). Body mass dependence of H+ leak in mitochondria and its relevance to metabolic rate. *Nature*, *362*(6421), 628–630. 8385274 10.1038/362628a0

[31] Boël, M., Romestaing, C., Duchamp, C., Veyrunes, F., Renaud, S., Roussel, D., & Voituron, Y. (2020). Improved mitochondrial coupling as a response to high mass-specific metabolic rate in extremely small mammals. *The Journal of Experimental Biology*, *223*(5), jeb215558. 32041806 10.1242/jeb.215558

[32] Henein, M. Y., Vancheri, S., Longo, G., & Vancheri, F. (2022). The role of inflammation in cardiovascular disease. *International Journal of Molecular Sciences*, *23*(21), 12906. 36361701 10.3390/ijms232112906 PMC9658900

