# Supplementary material for: Saccharomyces cerevisiae as a Model for Reprogramming of Eukaryotic Cells: Implications for the Study of the Relationship Between Metabolism and Inflammation in Chronic Disease
Source: Cell Biochem Biophys. 2025 Aug 18;83(4):5271–9. doi: 10.1007/s12013-025-01844-w (PMC12680766; doi:10.1007/s12013-025-01844-w)
Supplement: Supplementary file 1 — Supplementary Material [file 12013_2025_1844_MOESM1_ESM.docx]

# **Supplementary Material**


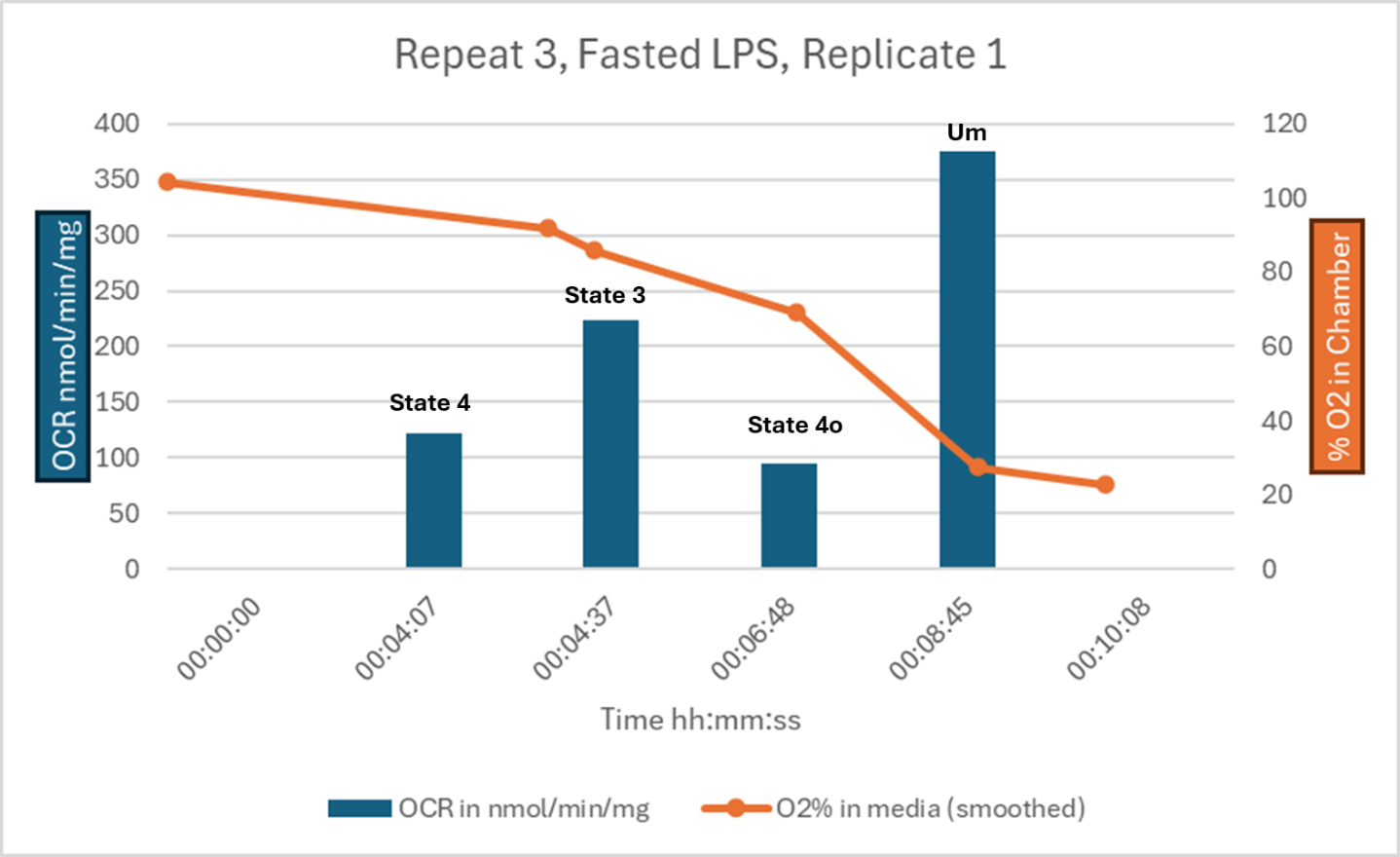


**(Sup Figure 1)** A reading from one of the samples to illustrate method: The %O_2_ trace starts when the mitochondrial prep was added to the closed chamber already containing the electrode buffer and D-Lactate. State 4 (without OMY) is the oxygen consumption rate prior to the addition of ADP seen as the first inflection in the % O_2_, State 3 after ADP was added indicates the OCR up until the second inflection when the oligomycin was added, State 4_O_ is after addition of oligomycin, U_m_ indicates Uncoupled Maximum OCR after FCCP additions seen as the steepest slope in %O_2_, ROX is determined residual OCR after Antimycin A addition seen after the final inflection..

**
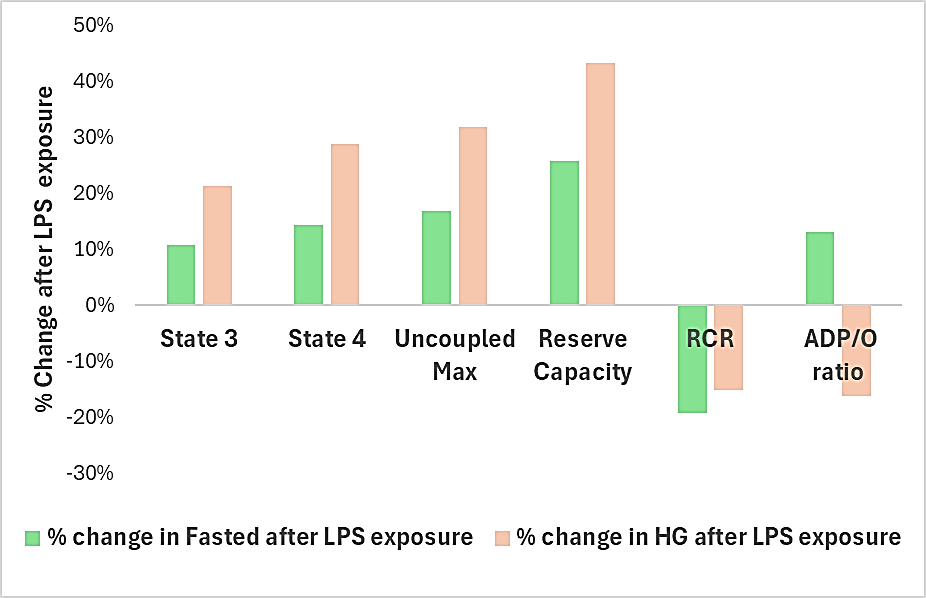
**

**Sup Figure 2:** **The changes in mitochondrial respiration responses (as a %).** Comparisons are between nutritional interventions (Fasted and HG), in response to being incubated for 90 minutes in HG broth containing LPS (100ng/ml) immediately prior to isolation.

**Sup Table 1: The changes in mitochondrial respiration responses (as a %), between nutritional interventions (Fasted and HG), in response to being incubated for 90 minutes in HG broth containing LPS** **(100ng/ml) immediately prior to isolation**. The value of the difference in the % change across the two nutritional interventions, considered here to be a reprogramming effect, is reported. P>0.10, less likely to be statistically significant, are highlighted in yellow. To determine statistical significance the following steps were taken: Values from the independent repeats were divided by the mean of the comparison data and multiplied by 100, before 1-tailed Student T tests were performed (paired when comparing data from the same conditioning and unpaired, assuming equal variance, when comparing across the two conditions).


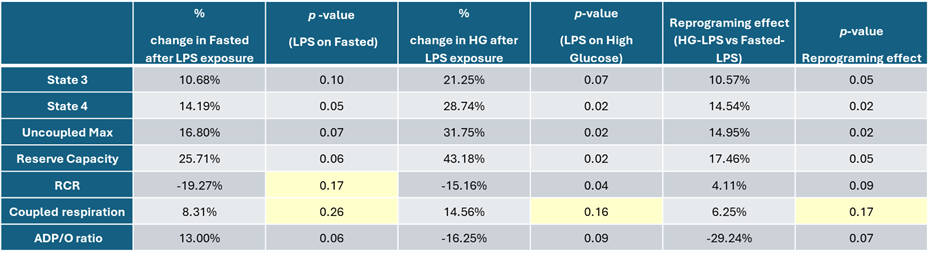


**References**

1. Howard JC. Introduction: cell-autonomous immunity. Microbes Infect. 2007 Nov 1;9(14):1633–5.

2. Furman D, Campisi J, Verdin E, Carrera-Bastos P, Targ S, Franceschi C, et al. Chronic inflammation in the etiology of disease across the life span. Nat Med. 2019 Dec;25(12):1822–32.

3. Chen L, Deng H, Cui H, Fang J, Zuo Z, Deng J, et al. Inflammatory responses and inflammation-associated diseases in organs. Oncotarget. 2017 Dec 14;9(6):7204–18.

4. Tiwari MrPS. Basic Mechanism Involved in the Process of Inflammation and Repair-I. In: Gupta DrSP, Gupta DrM, editors. Edited Book of Pathophysiology [Internet]. First. Iterative International Publishers, Selfypage Developers Pvt Ltd; 2024 [cited 2025 May 26]. p. 32–44. Available from: https://www.iipseries.org/viewpaper.php?pid=6468&pt=basic-mechanism-involved-in-the-process-of-inflammation-and-repair-i

5. Sun L, Yang X, Yuan Z, Wang H. Metabolic Reprogramming in Immune Response and Tissue Inflammation. Arterioscler Thromb Vasc Biol. 2020 Sep;40(9):1990–2001.

6. Ge T, Yang J, Zhou S, Wang Y, Li Y, Tong X. The Role of the Pentose Phosphate Pathway in Diabetes and Cancer. Front Endocrinol. 2020 Jun 9;11:365.

7. Fajgenbaum DC, June CH. Cytokine Storm. N Engl J Med. 2020 Dec 2;383(23):2255–73.

8. Nie J, Zhou L, Tian W, Liu X, Yang L, Yang X, et al. Deep insight into cytokine storm: from pathogenesis to treatment. Signal Transduct Target Ther. 2025 Apr 16;10:112.

9. Heinz A, Nonnenmacher Y, Henne A, Khalil MA, Bejkollari K, Dostert C, et al. Itaconate controls its own synthesis via feedback-inhibition of reverse TCA cycle activity at IDH2. Biochim Biophys Acta BBA - Mol Basis Dis. 2022 Dec 1;1868(12):166530.

10. Azzu V, Parker N, Brand MD. High membrane potential promotes alkenal-induced mitochondrial uncoupling and influences adenine nucleotide translocase conformation. Biochem J. 2008 Jul 15;413(2):323–32.

11. Kagan VE, Tyurin VA, Jiang J, Tyurina YY, Ritov VB, Amoscato AA, et al. Cytochrome c acts as a cardiolipin oxygenase required for release of proapoptotic factors. Nat Chem Biol. 2005 Sep;1(4):223–32.

12. Gardner PR, Fridovich I. Inactivation-reactivation of aconitase in Escherichia coli. A sensitive measure of superoxide radical. J Biol Chem. 1992 May;267(13):8757–63.

13. Engeham S, Mdaki K, Jewell K, Austin R, Lehner AN, Langley-Evans SC. Mitochondrial respiration is decreased in rat kidney following fetal exposure to a maternal low-protein diet. J Nutr Metab [Internet]. 2012 [cited 2020 Aug 27];2012. Available from: https://pubmed.ncbi.nlm.nih.gov/22536494/

14. Di Bartolomeo F, Malina C, Campbell K, Mormino M, Fuchs J, Vorontsov E, et al. Absolute yeast mitochondrial proteome quantification reveals trade-off between biosynthesis and energy generation during diauxic shift. Proc Natl Acad Sci U S A. 2020 Mar 31;117(13):7524–35.

15. Warburg O. The Metabolism of Carcinoma Cells1. J Cancer Res. 1925 Mar 1;9(1):148–63.

16. Matus-Ortega MG, Cárdenas-Monroy CA, Flores-Herrera O, Mendoza-Hernández G, Miranda M, González-Pedrajo B, et al. New complexes containing the internal alternative NADH dehydrogenase (Ndi1) in mitochondria of Saccharomyces cerevisiae. Yeast Chichester Engl. 2015 Oct;32(10):629–41.

17. Bonnefoy N, Fox TD. Directed Alteration of Saccharomyces cerevisiae Mitochondrial DNA by Biolistic Transformation and Homologous Recombination. In: Leister D, Herrmann JM, editors. Mitochondria: Practical Protocols [Internet]. Totowa, NJ: Humana Press; 2007 [cited 2023 Mar 27]. p. 153–66. (Methods in Molecular Biology^TM^). Available from: https://doi.org/10.1007/978-1-59745-365-3_11

18. Marques JM, Rodrigues RJ, de Magalhães-Sant’Ana AC, Gonçalves T. Saccharomyces cerevisiae Hog1 Protein Phosphorylation upon Exposure to Bacterial Endotoxin*. J Biol Chem. 2006 Aug 25;281(34):24687–94.

19. Gregg C, Kyryakov P, Titorenko VI. Purification of mitochondria from yeast cells. J Vis Exp [Internet]. 2009 [cited 2021 Mar 12];30(30). Available from: /pmc/articles/PMC3149909/

20. Reynafarje B, Costa LE, Lehninger AL. O2 solubility in aqueous media determined by a kinetic method. Anal Biochem. 1985 Mar;145(2):406–18.

21. Esteves TC, Parker N, Brand MD. Synergy of fatty acid and reactive alkenal activation of proton conductance through uncoupling protein 1 in mitochondria. Biochem J. 2006 Apr 11;395(3):619–28.

22. Pallotta ML, Valenti D, Iacovino M, Passarella S. Two separate pathways for d-lactate oxidation by *Saccharomyces cerevisiae* mitochondria which differ in energy production and carrier involvement. Biochim Biophys Acta BBA - Bioenerg. 2004 Feb 15;1608(2):104–13.

23. Brand MD, Nicholls DG. Assessing mitochondrial dysfunction in cells. Biochem J. 2011 Apr 15;435(2):297–312.

24. Affourtit C, Wong HS, Brand M. Measurement of Proton Leak in Isolated Mitochondria. In: Methods in molecular biology (Clifton, NJ). 2018. p. 157–70.

25. Morales-García L, Uribe-Carvajal S, Chiquete-Felix N, Espinoza-Simon E. The Reversible Opening of ScMuc Demonstrates a High Potential as a Cellular Protection System. Biophys J. 2019 Feb 15;116(3):419a.

26. Coupling/Uncoupling Reversibility in Isolated Mitochondria from Saccharomyces cerevisiae [Internet]. [cited 2025 Jul 16]. Available from: https://www.mdpi.com/2075-1729/11/12/1307

27. Brand MD, Pakay JL, Ocloo A, Kokoszka J, Wallace DC, Brookes PS, et al. The basal proton conductance of mitochondria depends on adenine nucleotide translocase content. Biochem J. 2005 Dec 1;392(Pt 2):353–62.

28. Azzu V, Parker N, Brand MD. High membrane potential promotes alkenal-induced mitochondrial uncoupling and influences adenine nucleotide translocase conformation. Biochem J. 2008 Jul 15;413(Pt 2):323–32.

29. Meng Q, Tian R, Long H, Wu X, Lai J, Zharkova O, et al. Capturing Cytokines with Advanced Materials: A Potential Strategy to Tackle COVID‐19 Cytokine Storm. Adv Mater Deerfield Beach Fla. 2021 May 20;33(20):2100012.

30. Dashko S, Zhou N, Compagno C, Piškur J. Why, when, and how did yeast evolve alcoholic fermentation? FEMS Yeast Res. 2014 Sep 1;14(6):826–32.

31. Porter RK, Brand MD. Body mass dependence of H+ leak in mitochondria and its relevance to metabolic rate. Nature. 1993 Apr;362(6421):628–30.

32. Boël M, Romestaing C, Duchamp C, Veyrunes F, Renaud S, Roussel D, et al. Improved mitochondrial coupling as a response to high mass-specific metabolic rate in extremely small mammals. J Exp Biol. 2020 Mar 11;223(5):jeb215558.

33. Henein MY, Vancheri S, Longo G, Vancheri F. The Role of Inflammation in Cardiovascular Disease. Int J Mol Sci. 2022 Jan;23(21):12906.
